# Development of a novel methyl cellulose hydrogel with physiologically relevant controlled ethanol release for cervical dysplasia ablation

**DOI:** 10.1002/btm2.70113

**Published:** 2026-01-21

**Authors:** Ashleigh M. Jankowski, Erela Imanoel, Gatha Adhikari, Jenna Mueller, Katharina Maisel

**Affiliations:** ^1^ Fischell Department of Bioengineering University of Maryland College Park Maryland USA; ^2^ Department of Obstetrics, Gynecology and Reproductive Science University of Maryland School of Medicine Baltimore Maryland USA; ^3^ Marlene and Stewart Greenebaum Cancer Center University of Maryland School of Medicine Baltimore Maryland USA

**Keywords:** cervical dysplasia, ethanol ablation, topical gel

## Abstract

Cervical cancer is a leading cause of death in women in low‐ and middle‐income countries (LMICs) and disproportionately affects women of minority populations in the United States, primarily due to a lack of infrastructural support for specialized care. A promising treatment that meets accessibility requirements is ethyl cellulose (EC)‐ethanol ablation—inducing necrotic cell death through application of ethanol to kill precancerous cells. While previous work focused on injecting EC‐ethanol to ablate high‐grade dysplasia (which can reach depths up to 5 mm below the tissue surface), low‐grade dysplasia requires a different delivery method as it is much more superficial (reaching depths of only 1–3 mm). Here, we have developed a topical gel for local ethanol ablation of low‐grade dysplasia with minimal damage to healthy cervical tissue. We investigated several gellants, including methyl cellulose (MC), EC, and Pluronic® F‐127, to develop an ethanol gel that meets parameters for low cost and topical ease of use. Formulations with F‐127 did not form gels with ethanol. Formulations with EC and MC were gel‐forming. The MC‐based formulations formed more uniform and stable gels that hold their own weight while still being spreadable at both room and body temperatures, key criteria for local cervical application. The optimal formulation contained 70% ethanol, 20% water, and 10% MC. One gram of this formulation represents approximately 5¢ material cost, and formulated gels were stable for at least 1 week when stored at 4, 22, 30, and 37°C. Additionally, the MC gel achieved localized ablation within 5 min after application to cervical cancer cells in vitro. Taken together, we have developed a low‐cost, efficacious, MC‐based ethanol gel fit for translational testing to treat low‐grade cervical dysplasia. This gel may provide a novel treatment option for women in LMICs, without causing major side effects or loss of healthy cervical tissue.

**Translational Impact Statement**

We have developed a low‐cost, efficacious, methyl cellulose‐based ethanol gel fit for translational testing to topically treat low‐grade cervical dysplasia. This addresses the need for a novel and accessible treatment option for women in low‐ and middle‐income countries, which will not cause major side effects or loss of healthy cervical tissue.

## 1 INTRODUCTION

Cervical cancer incidence is 2–3 times higher and mortality is 3–6 times higher in low‐ and middle‐income countries (LMICs) compared to high‐income countries (HICs).^1^, ^2^, ^3^, ^4^ Disparities in cervical cancer incidence and mortality also exist within HICs, with significant disparities occurring in populations with poor access to reproductive health services in the United States, most notably rural populations and historically marginalized racial groups.^5^, ^6^, ^7^, ^8^, ^9^ There is a need for better solutions across the cervical cancer care continuum, particularly in increasing access to treatment. For women diagnosed with low‐grade dysplasia (i.e., pre‐cancer), a “wait and see” approach is typically used in high‐income communities to see if lesions resolve on their own; however, in low‐income communities, women are often lost in the process of follow up care, which can allow for low‐grade dysplasia to develop into later stage cervical pre‐cancer or cancer. Because of this, the World Health Organization and others recommend “see‐and‐treat” approaches to enable cervical cancer prevention—that is, patients in low‐resource settings whose pre‐cancer are diagnosed and treated in the same day have better outcomes.^10^

In high‐income communities, high‐grade dysplasia is commonly treated through surgical excision. While effective, this approach removes substantial amounts of healthy cervical tissue and can lead to adverse obstetric outcomes and other adverse side effects.^11^ A more targeted alternative is ethanol ablation, which involves direct application of ethanol to malignant tissue, inducing cell death through necrosis.^12^ Ethanol ablation is particularly well‐suited for LMICs due to its low cost, portability, and ease of use.^13^ Promising pilot studies have demonstrated that combining ethanol with the gelling agent ethyl cellulose (EC) enhances local tissue necrosis while minimizing off‐target damage.^14^ While previous research has focused on EC‐ethanol injection for treating high‐grade dysplasia (which can extend up to 5 mm below the epithelial surface), low‐grade dysplasia is more superficial, typically confined to 1–3 mm depths, and therefore requires a different delivery strategy.^15^ To address this gap, we are developing a topical formulation of gel‐based ethanol ablation optimized for low‐grade dysplasia. This formulation would complement existing injectable treatments, enabling a more comprehensive therapeutic approach to cervical disease in LMICs. While several vaginal gel products exist—such as BufferGel and our own hypotonic thermogel—they are primarily water‐based and designed for small molecule delivery while preserving vaginal health. The addition of ethanol to these formulations disrupts their gel structure and adhesive properties. To overcome this limitation, we have developed a novel methyl cellulose–based ethanol gel that forms rapidly upon mixing and adheres to surfaces, including wet tissue phantoms. This formulation exhibits a well‐defined sol–gel transition and retains its gel‐like consistency at body temperature (37°C). It is also shear‐thinning, allowing for easy application using a custom‐designed applicator we propose to develop. Importantly, both the methyl cellulose matrix and ethanol therapeutic are low‐cost and widely available, making this solution well aligned with the resource constraints of LMICs. Many gels have been developed for topical vaginal drug delivery for controlled drug release. Typically formulated from water‐soluble polymeric materials such as xanthan gum, carboxymethyl‐cellulose, hydroxyethyl cellulose, or carbopols, these materials mix with cervicovaginal mucus in the vaginal canal and provide sustained drug delivery for 12–24 h.^16^ A number of gels have also been designed for treating cervical cancer through vaginal application, such as a thermosensitive polymer (e.g., Pluronic® F‐127) gel developed specifically to treat HPV‐induced cervical cancer,^17^ nanoparticle‐loaded and small‐molecule loaded poloxamer 188/407 (F‐127/F188)‐based temperature‐sensitive in situ gels,^18^, ^19^ and a retinyl‐acetate delivering gel.^20^ These gels generally deliver expensive drugs to the entire vaginal tissue (not specifically the cervix) and usually require repeated application, making them less suitable for treating low‐grade lesions in low‐resource settings. Some existing topical cancer treatments have been suggested for the off‐label treatment of cervical cancer, notably Imiquimod and 5‐fluorouracil creams.^21^ These creams, however, have largely been investigated for late‐stage dysplasia treatment, as they trigger intense physiological responses, which would be unduly aggressive for low‐grade dysplasia. Furthermore, these gels are water based and made for delivery of small molecule therapeutics; adding ethanol disrupts their solid‐like gel structure and stickiness, making adapting an existing formulation insufficient for ethanol ablation applications. As such, there is a need to develop an ethanol‐based gel specifically for cervical application for treatment of low‐grade dysplasia.

An ethanol‐based gel for topical ablation of low‐grade cervical dysplasia will need to have sufficient rheological properties for topical application: the ability to hold its own weight, maintain a storage modulus greater than loss modulus, and high (>100 Pa*s) viscosity maintained at both body temperature (37°C) and room temperature (22°C). Further, to meet accessibility requirements, the gel will need to maintain these properties after storage under varying conditions and for extended periods of time, while being low‐cost and of simple formulation. Finally, the gel must release ethanol at a rate relevant for achieving kill of cervical dysplasia cells. To develop this gel, we investigated multiple gellants, including Pluronic® F‐127, due to its popularity in literature for medical gels; ethyl cellulose (EC), due to its demonstrated capability to form ethanol‐releasing gels; and methyl cellulose (MC), due to its demonstrated ability to form ethanol‐based gels that maintain properties at varying temperatures, all of which are FDA‐approved generally regarded as safe (GRAS) materials,^13^, ^22^, ^23^, ^24^, ^25^, ^26^, ^27^ and, in the case of cellulose gellants, known to be very low‐cost.^28^, ^29^ We investigated storage and loss moduli and viscosity with these various formulations and found that 70% ethanol, 20% water, and 10% MC gel best exemplified goal parameters in that it is low‐cost and has sufficient viscoelastic properties to hold its own weight while maintaining spreadability for topical application. We then tested this gel for ethanol release and localized cervical cancer cell‐killing in vitro.

## 2 METHODS

### 2.1 Materials

Methyl cellulose (MC) viscosity 4000 cP (Thermo Fisher, Geel, Belgium) was received from Thermo Fisher Scientific and used without further purification. Ethyl Cellulose (EC) viscosity 100 cP and Pluronic® F‐127 (F127) Powder were acquired from Sigma Aldrich (Sigma‐Aldrich, Co., St. Louis, MO) and used without further purification. The 200 Proof Ethanol (EtOH) (200 proof, Pharmco) was the primary solvent used other than Millipore Ultrapure deionized (DI) water.

### 2.2 Gel formulation

All gel formulation was performed at room temperature. Gellant (MC, EC, or F127) was weighed out to the appropriate mass and slowly added to ethanol at desired concentrations (Table 1) while mixing with a stir bar or by hand with a spatula. Added EC required more time to dissolve than MC/F127, approximately 1 h. After adding the entire measured mass of gellant, gels were further mixed using a handheld tissue homogenizer. Gels were stored in 20 mL volume scintillation vials sealed with parafilm at room temperature to avoid ethanol evaporation. Except for specified stability studies, all gels were used within 1 week of formulation.

**TABLE 1:** Concentrations of gellants and solvents investigated.

Material	% Gellant (w/w)	% DI Water	% EtOH
MC	6%–15%	10%–20%	70%–79%
EC	6%–40%	‐	60%–94%
F127	5%–35%	20%–80%	71%–82%

### 2.3 Rheological analysis

Solution viscosity and rheological characterization was performed using an Ares G2 Rheometer (TA Instruments, New Castle, DE) equipped with a 40 mm diameter 1.991° stainless steel cone plate geometry and a solvent trap. Viscosity was evaluated through use of oscillation time sweep flow ramps, wherein shear rate was linearly decreased from 250 to 1 s^−1^ over 60 s followed by a reverse wherein the shear rate was linearly increased from 1 to 250 s^−1^ over 60 s. Storage and loss moduli were evaluated using an oscillation time sweep, wherein an angular frequency of 10 rad/s and a constant strain of 1% was applied over a duration of 240 s. The angular frequency of 10 rad/s and strain of 1% were chosen based on common use for similar gels in literature, as well as published analyses that methyl cellulose and related cellulose systems maintain linear viscoelastic behavior for strains <5%.^30^, ^31^, ^32^ All analyses were performed in triplicate at 22°C or 37°C. Any samples that were stored in a refrigerator for stability study purposes were also evaluated at 4°C. Formulations were defined as a successful gel if they met specific criteria: a storage modulus (G′) significantly greater than a loss modulus (G″), time‐dependent viscoelastic response under applied shear which showed a yield stress, and qualitatively able to hold their own weight (evaluated via inversion in scintillation vial) while still being spreadable (easily spread into a homogeneous even layer).^33^, ^34^, ^35^, ^36^

### 2.4 Release studies

Release studies were performed to evaluate the ethanol delivery capabilities of the developed gels. Gels were loaded into 10 mm flat width dialysis tubing cellulose membrane (Sigma Aldrich, St. Louis, MO) sealed with dialysis tubing clips (Spectrum™ Dialysis Tubing Closures: Spectra/Por™ Standard 12 mm), and submerged in 1X PBS at 37°C in a sealed container on a tube rotator (50 mL centrifuge tubes filled to reduce air volume, final PBS volume of 53 mL). 3 × 300 μL samples were taken at set time points over the course of 6 h. Ethanol concentration was detected using an Amplite® Ethanol Quantitation Kit (AAT Bioquest, Pleasanton, CA) and a Spark Multimode Microplate Reader.

### 2.5 Stability studies

Stability studies were performed by storing gels at various temperatures and evaluating the changes in rheology over time. Temperatures included 4°C, as the standard refrigeration temperature, 22°C, as average room temperature, 30°C, as the highest recorded average room temperature in sub‐Saharan African LMICs, and 37°C, to simulate high temperature outdoor conditions in sub‐Saharan African LMICs.^37^ These studies were designed to simulate real‐world storage and transport conditions, taking into account that resource‐poor settings often have limited access to climate‐controlled storage. A 10 mL volume of gel was prepared and stored in 20 mL scintillation vials, which were sealed. Gel mass and rheological characterization were recorded at set intervals—mass every day for a week and rheological characterization at completion of the trial period. The trial period was performed for 1 week in order to confirm that gels maintained stability from formulation time to use over the course of studies, as well as to begin validation for storage in a clinical setting (long‐term studies currently ongoing). Rheological characterization was performed at 22°C as described above. Gels were also qualitatively analyzed to assess if clumping or ethanol‐gellant separation occurred. Parameters to identify a gel as stable were viscosity peak, which is not significantly different from the identified average peak for that gel when made fresh, a storage modulus that is greater than loss modulus, no statistically significant changes in mass, and no observed gel separation.

### 2.6 In vitro cell killing assay

A human cervical cancer cell line, SiHa, from ATCC (ATCC® HTB‐35™, Manassas, VA) was used to determine ethanol killing. SiHa cells were expanded in Eagle's Minimum Essential Medium (EMEM, ATCC), supplemented with 10% FBS (Sigma‐Aldrich, St. Louis, MO) and 1% Penicillin–Streptomycin (P/S, Sigma‐Aldrich). Cells were expanded in standard cell culture conditions (37°C, 21% O_2_, 5% CO_2_) in six‐well plates. Cells were recovered at 80% confluency. A volume of 10 μL of the gel, 1X phosphate‐buffered saline (PBS) or 200 proof ethanol were added to the designated wells. Cells were exposed to gels/control for 5 min. This timescale was chosen as it is more than enough time for ethanol killing of a single cell layer,^38^ while still being short enough that cells can survive without addition of media, a potential vector for ethanol spread. Viability was then assessed with a live/dead assay by adding calcein AM (2 M, eBioscience™ Calcein AM Viability Dye, 65‐0853‐39, San Diego, CA) and propidium iodide (5 M Propidium Iodide Ready Flow™ Reagent, R37169) to the cells for 15 min. Cells were imaged at 10X using a Nikon Ti2 fluorescence microscope. Next, live and dead cells in each treatment group were counted in ImageJ Fiji (NIH, Bethesda MD). For each image live and dead channels were split, then the image was thresholded, and a binary image was obtained. Then the analyze particle tool was used and cells were counted with a manually created ROI. All studies were performed in triplicate.

## 3 RESULTS

### 3.1 F127 does not form gels in combination with ethanol

**FIGURE 1:**
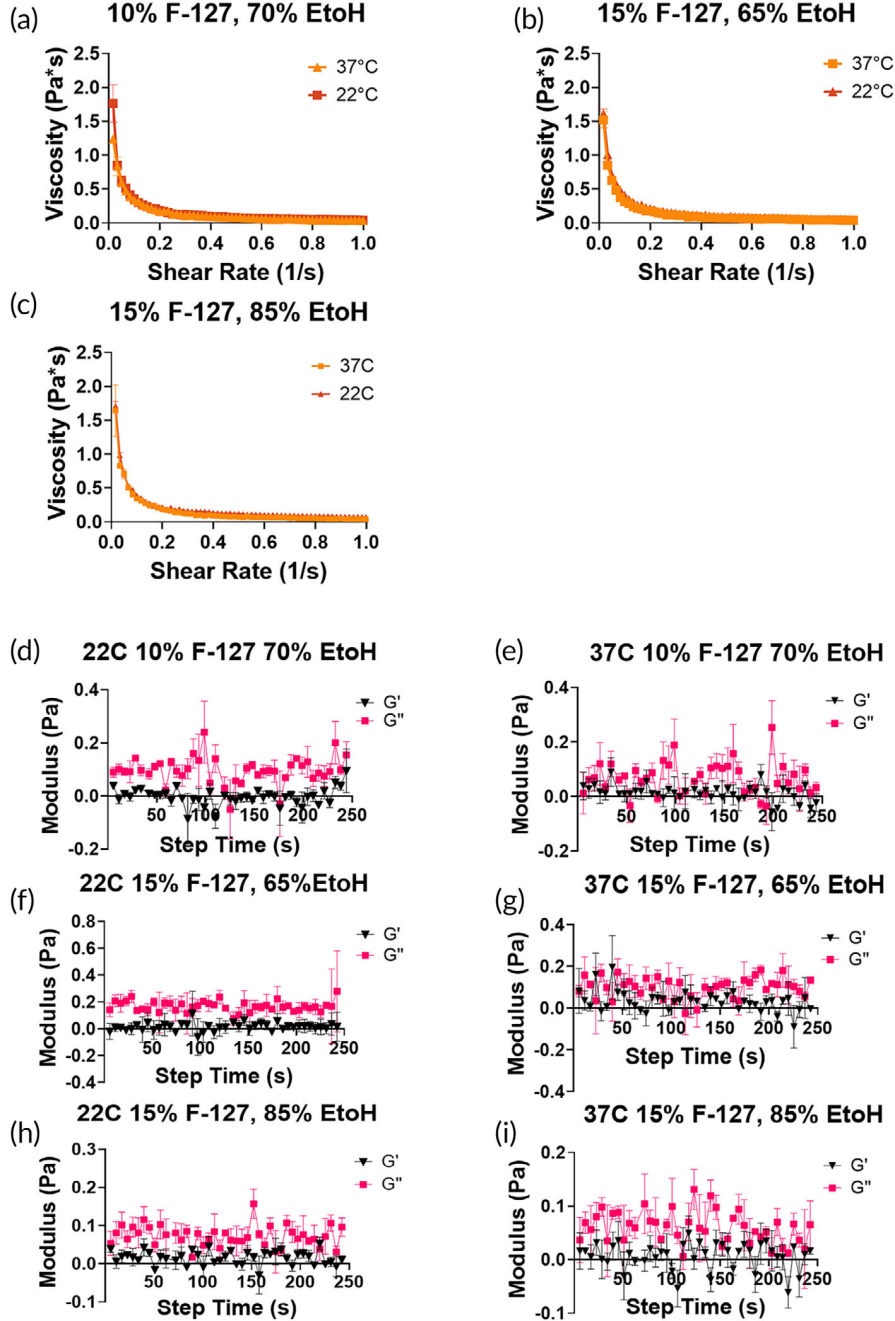
Rheological properties of Pluronic® F‐127 Gel formulations. Average ± SEM values for viscosity‐stress curves (top) and storage‐loss moduli (bottom). Data shown for formulations of 10% Pluronic® F‐127, 20% water, 70% ethanol (A, D, E), 15% Pluronic® F‐127, 20% water, 65% ethanol (B, F, G), 15% Pluronic® F‐127 and 85% ethanol (C, H, I). *N* = 3 for all formulations.

F127 as a gellant for ethanol‐based gels was evaluated across a range of concentrations (5%–35% [w/w]). Concentrations lower than 15% did not form gel‐like materials at room temperature, which aligns with literature understanding of F127 critical gelling concentrations,^39^ and concentrations higher than 25% tended to approach more rubbery, solid‐like textures. F127 combined with 200 proof ethanol alone did not form a homogeneous mixture; the components stayed separated. Gels that successfully formed homogeneous formulations were evaluated for viscosity and storage/loss moduli. For all formulations and temperatures, viscosity peaks were less than 10 Pa*s, indicating that the formulations were not forming a gel: the maximum viscosity achieved in independent F127 gels was the 10% F127, 20% water, 70% ethanol formulation at 0.054 ± 0.007 Pa*s. The 15% F127, 20% water, 65% ethanol formulation peaked at 2.7 Pa*s; 15% F127, 85% ethanol peaked at 1.9 Pa*s (Figure 1a–c). These peaks also occurred at the beginning of a viscosity shear sweep, after which the viscosity drastically decreased for all, indicating shear‐thinning behavior. For all evaluated formulations and temperatures containing ethanol (15% F127, 20% water, 65% ethanol; 15% F127, 85% ethanol; 10% F127, 20% water, 70% ethanol; at 37°C and 22°C), the loss modulus was greater than the storage modulus, indicating the formulations behaved more like a viscoelastic liquid than a gel (Figure 1d–i). Additionally, the moduli did not stay the same when at both 22°C and 37°C for all formulations, indicating a temperature‐dependent viscoelastic profile. The only F127 formulation that formed a successful gel was that which did not include ethanol, the 20% F127, 80% water formulation. This formulation displayed viscosity above 3900 Pa*s for all temperatures and a storage modulus greater than loss modulus for all temperatures (Figure S1A–C). Overall, F127 gels lacked the properties necessary for sufficient gelation: ability to hold their own weight, time‐dependent viscoelastic response under applied shear which showed a yield stress, and inability to maintain gel properties at room and body temperatures.

### 3.2 High percentage ethyl cellulose formulations formed gels and exhibited temperature‐dependent properties

**FIGURE 2:**
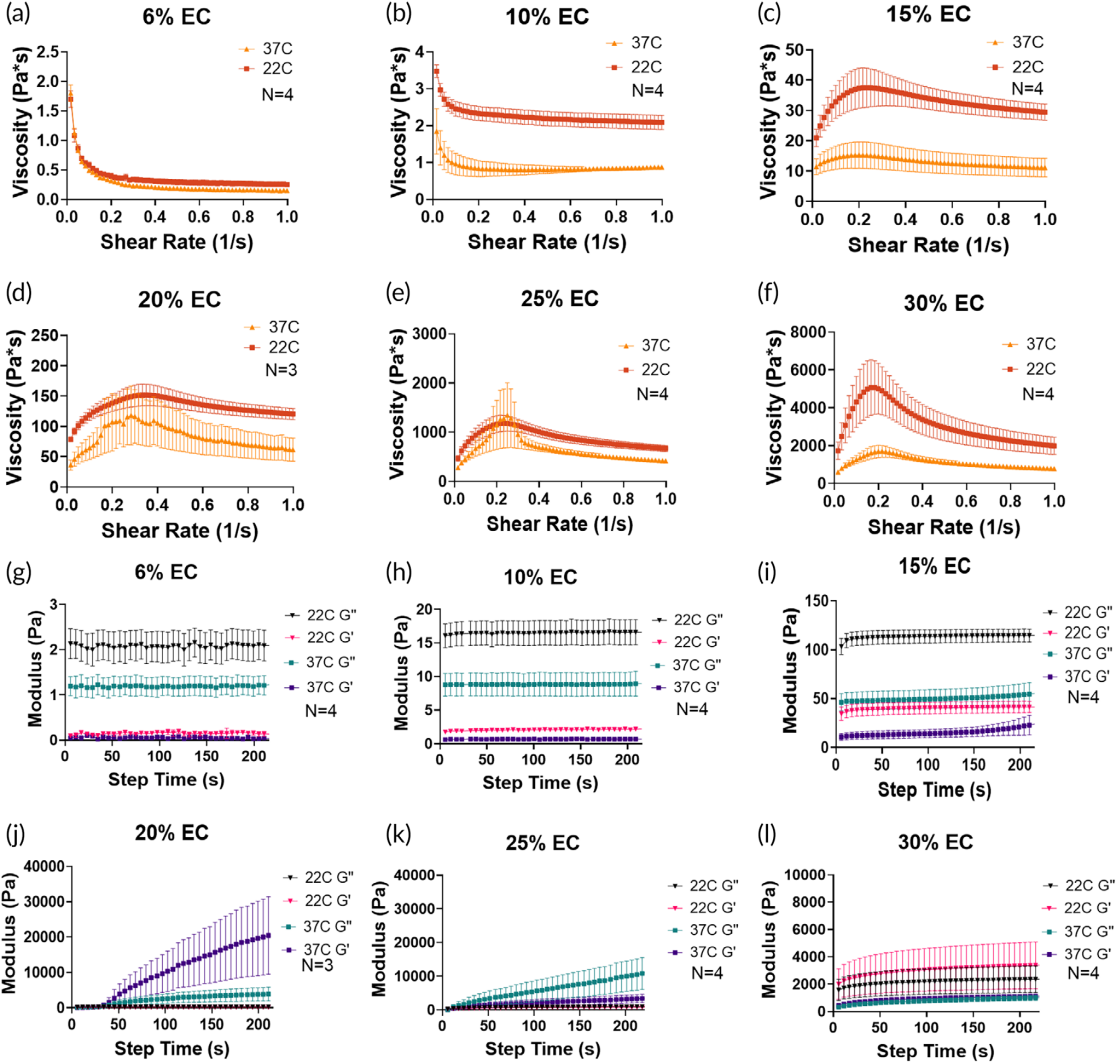
Rheological properties of ethyl cellulose gel formulations from 6% to 30% EC. Average ± SEM values for viscosity‐stress curves at 22°C and 37°C (a–f), and storage and loss modulus oscillatory time sweeps (g–l).

Ethyl cellulose (EC) formulations were made with 6, 10, 15, 20, 25, 30, and 40% EC and ethanol. The viscosity of these formulations greatly decreased when brought up to 37°C, with little to no peaks, suggesting these formulations lost their gel properties at high temperatures (i.e., they were melting) (Figure 2a–f). At EC percentages less than 30%, the loss modulus was greater than the storage modulus at both 22°C and 37°C, indicating the formulations were a viscoelastic liquid as opposed to a gel (Figure 2g–l). The 40% EC formulation was a non‐spreadable near‐solid at room temperature. 40% EC data not shown, as formulations were so stiff they were a malleable solid instead of a gel, making rheological testing in the same manner as was done for 6–40% gels nonviable.

### 3.3 Methyl cellulose formulations formed temperature‐stable high‐viscosity gels

Methyl cellulose (MC) formulations were tested at a broad range of MC, water, and ethanol concentrations to identify the maximum and minimum concentrations that could be used to formulate a homogeneous solution. Mixtures with >10% MC and/or <20% water had incomplete dissolution of MC and did not form a homogeneous gel. Mixtures with >75% ethanol tended to separate. The MC formulations within these ranges at all concentrations had storage moduli (G′) significantly greater than loss moduli (G″) (Table 2). However, only certain formulations exhibited a time‐dependent viscoelastic response under applied shear, which showed a yield stress (Table 2). Formulations which exhibited a yield stress and had G' > G″ were considered to have formed a viable gel.

**TABLE 2:** Methyl cellulose formulations rheological data.

% MC	% Water	% EtOH	Exhibited yield stress?	G′ > G″ at 37C?	Maximum viscosity at 22°C (Pa*s)	Maximum viscosity at 37°C (Pa*s)	Formed gel?
6	20	74	No	Yes	5550	15,240	No
6.5	15.5	76	No	Yes	2698	17,670	No
8	13	79	Yes	Yes	15,480	73,240	Yes
9	14	77	No	Yes	18,140	18,250	No
	17	74	No	Yes	8807	10,550	No
	14	77	No	Yes	18,140	18,250	No
9.5	14.5	76	No	Yes	38,690	32,080	No
	18.5	72	No	No	7884	14,700	No
	14.5	76	No	Yes	38,690	32,080	No
	18.5	72	No	Yes	7884	14,710	No
	14	76.5	Yes	Yes	51,220	27,720	Yes
	15	75.5	No	No	788.7	590.2	No
	14.5	76	No	Yes	38,690	32,080	No
10	20	70	Yes	Yes	2558	2265	Yes
	14.5	75.5	Yes	Yes	526.9	3013	Yes
	15	75	Yes	Yes	1129	2157	Yes
15	10	75	Yes	Yes	5600	981.3	Yes

**FIGURE 3:**
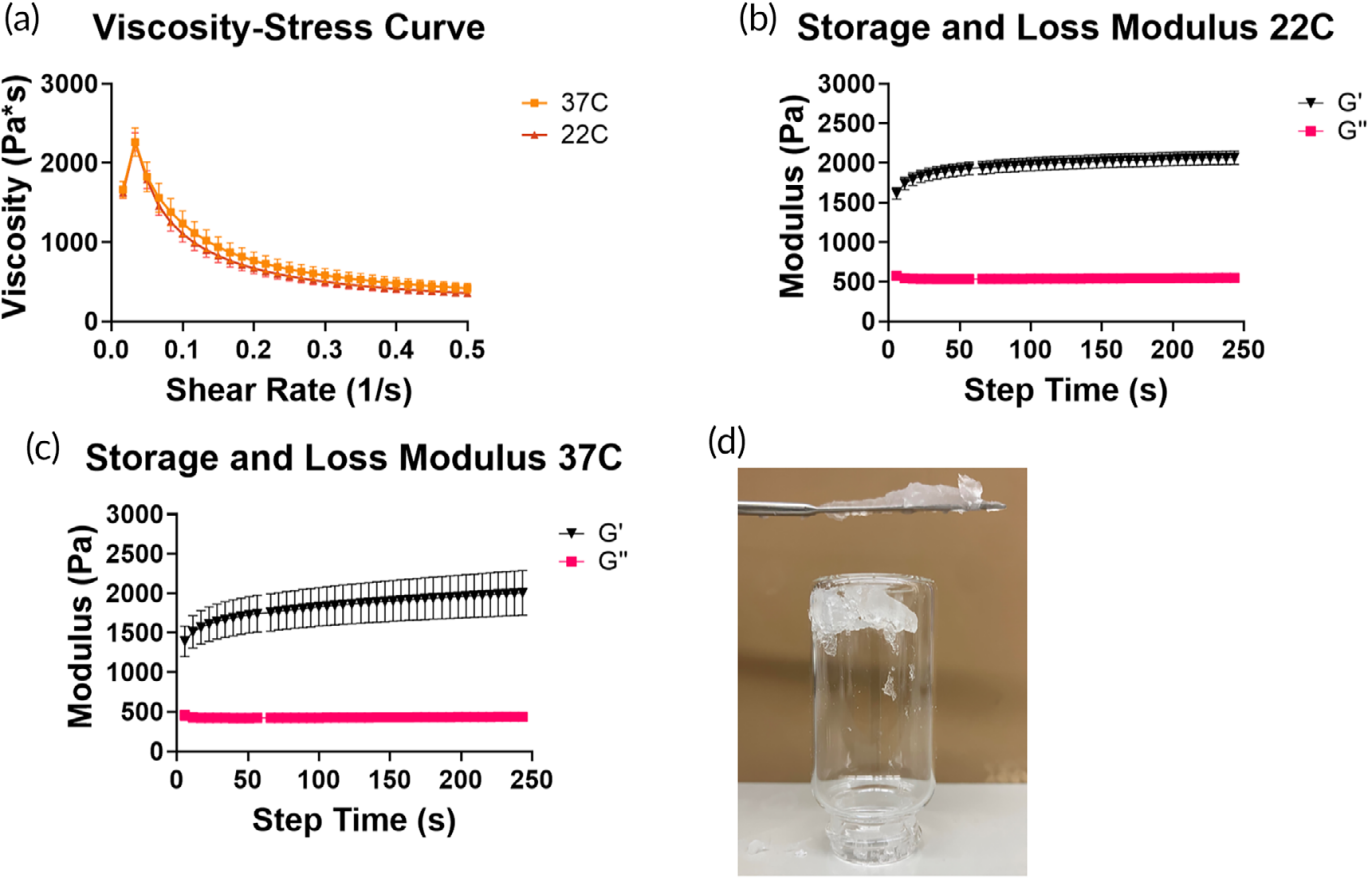
Average rheological data for 20% MC, 10% water, 70% ethanol formulation. Average ± SEM values for viscosity‐stress curve (a) and storage and loss modulus over oscillatory time sweep at (b) 22°C and (c) 37°C. Representative of *N* = 8+. (d) Representative image of gel holding its own weight on spatula and in inverted scintillation vial.

The formulation with the most consistency in measurements and that qualitatively had texture best able to be spread into an even layer while still holding its own weight was the 10% MC, 20% water, 70% ethanol formulation. This gel had viscosity peaks between 2000 and 2500 Pa*s at both 22°C and 37°C (Figure 3a). A peak indicating yield stress is exhibited at 74.9 Pa, and post‐yield stress as the shear rate increases, viscosity declines and stabilizes between 200 and 270 Pa*s (Figure 3a). Across both 22°C and 37°C, G′ consistently exceeded G″ by a factor of >3–4, with no significant difference between rheological measurements (Figure 3b,c) and the gel formed a homogeneous mixture that holds its own weight (Figure 3d).

### 3.4 The 10% MC gels release 70% ethanol content after 3 h and gels are stable for at least 1 week

**FIGURE 4:**
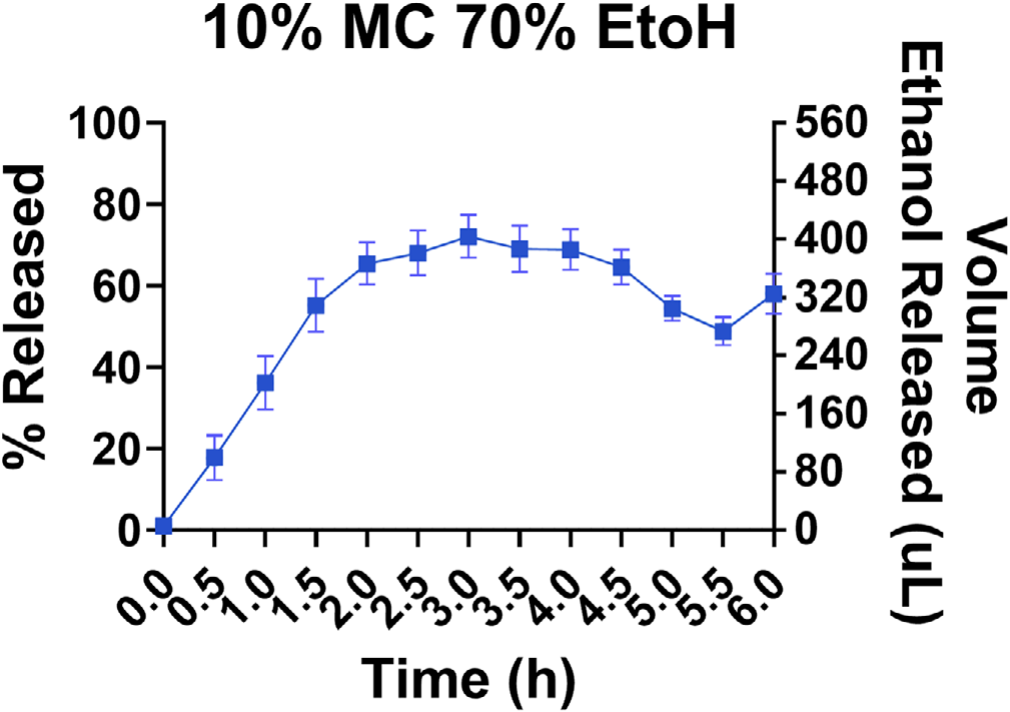
Release over time of ethanol from 10% MC, 20% water, and 70% ethanol gel into PBS. Average ± SEM values for release of ethanol from 1130 mg of 10% MC, 20% water, and 70% ethanol gel formulation in PBS at 37°C. *N* = 3.

Ethanol release for the formulation with optimal rheological properties (10% MC, 20% water, 70% ethanol) occurred in <4 h, after which equilibrium was reached (Figure 4). Approximately 72% ± 16% of the loaded ethanol was released, indicating high efficacy for controlled ethanol release. A dip in percentage released was observed around 5 h, likely due to evaporation of ethanol from the sink after repeated opening and resealing of the container.

**FIGURE 5:**
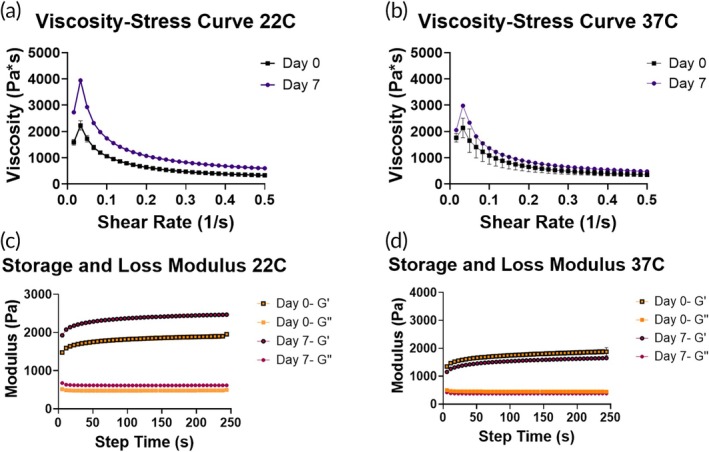
One‐week stability study rheology data. Average ± SEM values viscosity stress curves at 22°C (a) and 37°C (b) and storage and loss moduli at 22°C (c) and 37°C (d) for 10% MC, 20% water, 70% ethanol gel stored at room temperature on days 0 and 7. *N* = 3.

The formulation maintained gel properties over the course of 1 week. When stored at room temperature for 7 days, yield stress viscosity peak was maintained above 2000 Pa*s (Figure 5a,b) and storage modulus was still greater than loss modulus when evaluated at both 22°C and 37°C on the seventh day (Figure 5c,d). Samples taken throughout the week on days 3 and 5 to verify consistency showed similar trends (Figure S2A–D).

### 3.5 MC‐ethanol gel locally kills SiHa cells within 5 min of gel exposure

**FIGURE 6:**
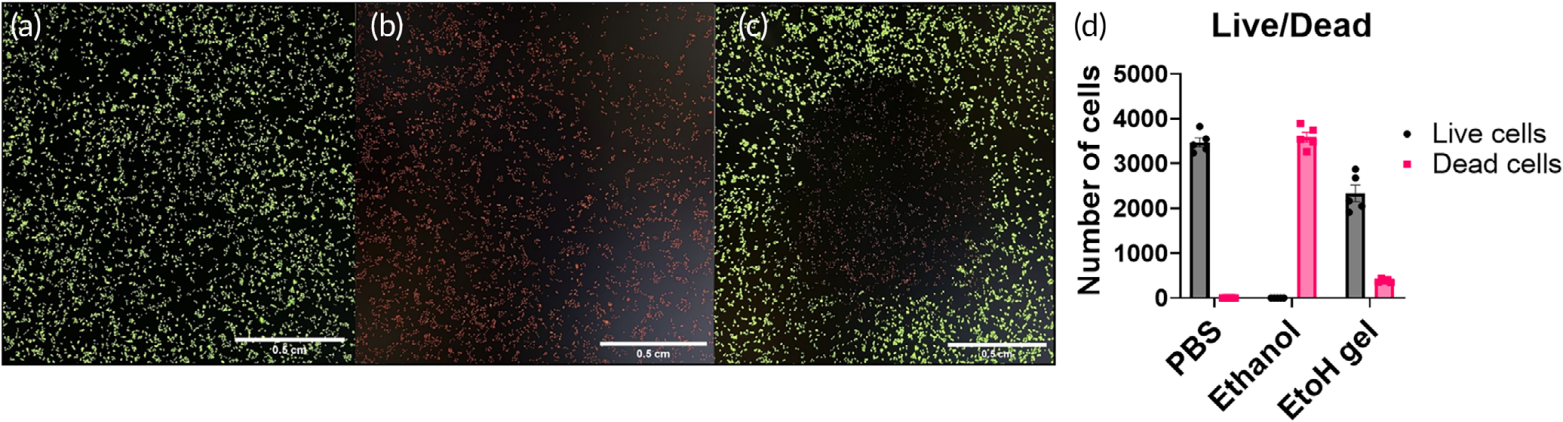
Cell viability outcomes in SiHa monolayers. Microscopy images (a–c) and total cell number (d). Average ± SEM values, *N* = 5.

The developed gel was then evaluated for cell killing abilities on a human cervical squamous cell carcinoma cell (SiHa) monolayer. Images of SiHa monolayers revealed differential cell viability outcomes based on the applied solution (Figure 6a). The PBS control showed no observable cell death (Figure 6a). The ethanol‐only control (70% ethanol without MC) led to complete cell death across the treated area (Figure 6b). MC gel (10% MC, 20% water, 70% ethanol) resulted in an average of 388 dead cells out of 2722 total and a total cell death area of 0.785 cm^2^ out of 3.06 cm^2^ total area. (Figure 6c,d) shows cell death in the area where the gel was applied while leaving the surrounding cells alive. With MC gel, cell kill was observed in 25% of the total area, whereas the PBS control had 0% cell kill and ethanol had 100% cell kill.

## 4 DISCUSSION

Current ethanol ablation techniques for tumors primarily rely on direct ethanol injection into the lesion. While effective for some tumor types, these methods are not suitable for treating low‐grade cervical dysplasia, as they pose a significant risk to surrounding healthy tissue.^40^ Previous work successfully developed an injectable ethanol gel for the ablation of high‐grade cervical dysplasia, offering a low‐cost and targeted treatment capable of inducing sufficient necrosis while preserving adjacent healthy tissue.^22^ However, a holistic cervical cancer prevention strategy for LMICs also requires a safe and effective approach for treating low‐grade dysplasia, which is more superficial, typically confined to depths of 1–3 mm.^15^ To address this, we are developing a topical ethanol gel specifically designed to kill tissue only at the site of application, sparing the rest of the cervical surface. This contrasts with current clinical methods such as LEEP, cryotherapy, and thermocoagulation, which often treat the entire cervical face, leading to overtreatment and increased risk of complications. The previously developed injectable gel is not suitable for topical use, as its rheological properties do not allow it to hold its own weight or adhere well when applied to a surface. In contrast, our newly developed gel formulation possesses the mechanical properties needed to remain in place on wet tissue surfaces, delivering localized ablation without specialized equipment. This innovation holds the potential to significantly improve clinical outcomes for women in LMICs and other resource‐limited settings. Of the gellants investigated, only MC was found to form an ethanol‐based gel that maintained its properties at room and body temperatures. The F127 and EC gels containing ethanol failed to gel and/or maintain consistent properties at both temperatures, making these gellants nonviable for treating low‐grade cervical dysplasia lesions locally. The MC formulation which best exhibited the required properties was the formulation containing 10% MC, 20% water, and 70% ethanol. This finding is consistent with similar gels of slightly varied ethanol and methyl cellulose concentrations, which have also demonstrated extreme resistance to temperature change.^25^ At all temperatures, this formulation exhibited high viscosity as well as time‐dependent viscoelastic response under applied shear which showed a yield stress, has a storage modulus greater than loss modulus (indicating a viscoelastic solid and thus gel formation), was spreadable, and held its own weight, properties which align with those defined for medical use semisolids,^41^ and these properties were maintained at both room and body temperature. This is unique from most common high‐ethanol content (>70%) gels, which are typically used for topical bactericide and virucide and as such are less viscous and do not retain gel properties at all temperatures.^42^, ^43^

Release of ethanol from the gel was evaluated to provide insights into whether the gel will release ethanol at a rate relevant for cell‐kill under physiological conditions. The release of 72% of the loaded ethanol over the course of 4 h indicates a slow release of the majority of the loaded ethanol, which is successful for controlled, local cell killing. Further, the volume of ethanol released from the gel aligns with the volume of ethanol needed to achieve tumor killing in other models.^13^ The volume of ethanol required for tumor cell kill is proportional to the tumor size, with tumors less than 2.5 cm in size requiring as low as 0.1 mL ethanol to achieve cell kill according to current literature.^44^ Given that the average size of cervical dysplastic regions at diagnosis is 6.2 mm^45^, it can be approximated that less than 115 mg of our topically applied gel would be needed for ablation of these lesions. Other developed gels which aim to address cervical cancer topically via release of small molecule drugs tend to require larger doses (up to 2.5 g) and repeat administration (i.e., weekly for 10 weeks),^46^, ^47^ which would not meet see‐and‐treat requirements. Further, 115 mg of gel represents less than one‐cent worth of material costs, sufficiently surpassing The World Bank's requirements for cost‐effective interventions for LMICs, defined as less than 100 USD per year of life saved.^48^ The gel was additionally evaluated for accessibility requirements through its ability to maintain gel properties after a week‐long storage at various temperatures, representative of refrigeration storage and room temperature storage in various climates. The gel maintained its properties in all storage conditions, with little to no loss of ethanol due to evaporation or separation during the storage period.

Our optimal gel formulation was shown to release volumes of ethanol at a controlled rate, which will be significant for cell kill. The gel enables controlled cell kill in SiHa monolayers by modulating ethanol diffusion. The application of the MC gel (10% MC, 20% water, 70% ethanol) resulted in partial cell death, with an average of 388 cells affected within an area of 0.785 cm^2^, only in the applied region of interest. In contrast, the ethanol‐only control led to complete cell death across the treated area, indicating that ethanol alone rapidly diffuses and causes widespread cytotoxicity. The ability of the gel to control ethanol release into only areas of direct application further verifies its viability as a treatment, as this will significantly limit adverse off‐target effects, a critical piece of design criteria for cervical dysplasia treatments.^49^ Existing mechanisms for SiHa ablation have comparable monolayer cell kill as the free ethanol group, including application of DOX‐loaded AuNR micelles with NIR irradiation^50^ and MoS2‐gold nanoparticle supporting nanoribbons for photothermal ablation.^51^ This area‐controlled cell death suggests that the MC gel acts as a vital diffusion barrier, slowing ethanol release and limiting its cytotoxic effects. Additionally, this aligns with current understandings of cellulose‐based gels with added ethanol components, in that the addition of ethanol enhances tissue penetration depth while cellulose acts as a controlling factor, aiding in targeted cancer cell killing.^52^ This controlled release could be advantageous in applications where partial cell elimination is required, such as targeted therapy or tissue engineering. Future investigation is needed over a longer time scale and in a more complex model to evaluate the penetration depth capabilities of the gel. By preventing excessive ethanol exposure, the MC gel may help preserve surrounding healthy cells while still achieving desired localized cell kill.

## 5 CONCLUSION

The work described herein outlines the development of a novel topical gel formulation for cervical dysplasia ablation. This gel of 10% MC, 20% water, and 70% ethanol is novel in terms of its formulation, properties, and biomedical applications. It has the ability to hold its own weight and the viscoelastic properties of a gel, making it viable for topical applications, a property which it maintains at both room and body temperature. The gel also releases ethanol at a clinically relevant rate causing significant cancer cell death in low‐grade cervical dysplasia, indicating significant promise for tumor‐ablative properties. The gel further meets requirements for see‐and‐treat methodology in resource‐poor settings such as LMICs and rural localities—inexpensive, shelf stable at a variety of temperatures, and requiring no specialized equipment for use. Future work needs to be done to investigate the efficacy of the gel on a tumor model in vitro beyond just the monolayer, as well as in vivo investigations.

## AUTHOR CONTRIBUTIONS

**Ashleigh M. Jankowski:** Conceptualization; investigation; writing – original draft; methodology; validation; writing – review and editing; formal analysis; project administration; data curation; supervision. **Gatha Adhikari:** Investigation; writing – original draft; writing – review and editing.

## FUNDING INFORMATION

Primary funding for the project was startup funds provided by the University of Maryland.

## CONFLICT OF INTEREST STATEMENT

The authors declare no conflicts of interest.

## Supporting information


**Figure S1.** Rheological properties of 20% Pluronic F‐127 80% water formulation. Average ± SEM values for viscosity shear sweep (A) and storage and loss modulus data at 22 (B) and 37C (C). *N* = 3.
**Figure S2.** Day 3 and 5 rheology data from one‐week stability study. Average ± SEM values viscosity stress curves at 22C (A) and 37C (B) and storage and loss moduli at 22C (C) and 37C (D) for 70% ethanol 10% MC 20% H2O gel stored at room temperature. *N* = 3.

## Data Availability

The data that support the findings of this study are available from the corresponding author upon reasonable request.
