# Supplementary material for: Development of a novel methyl cellulose hydrogel with physiologically relevant controlled ethanol release for cervical dysplasia ablation
Source: Bioeng Transl Med. 2026 Jan 21;11(3):e70113. doi: 10.1002/btm2.70113 (PMC13247434; doi:10.1002/btm2.70113)
Supplement: Supplementary file 1 — Figure S1. Rheological properties of 20% Pluronic F‐127 80% water formulation. Average ± SEM values for viscosity shear sweep (A) and storage and loss modulus data at 22 (B) and 37C (C). N = 3. Figure S2. Day 3 and 5 rheology data from one‐week stability study. Average ± SEM values viscosity stress curves at 22C (A) and 37C (B) and storage and loss moduli at 22C (C) and 37C (D) for 70% ethanol 10% MC 20% H2O gel stored at room temperature. N = 3. [file BTM2-11-e70113-s001.docx]

**Supplemental Materials**

**
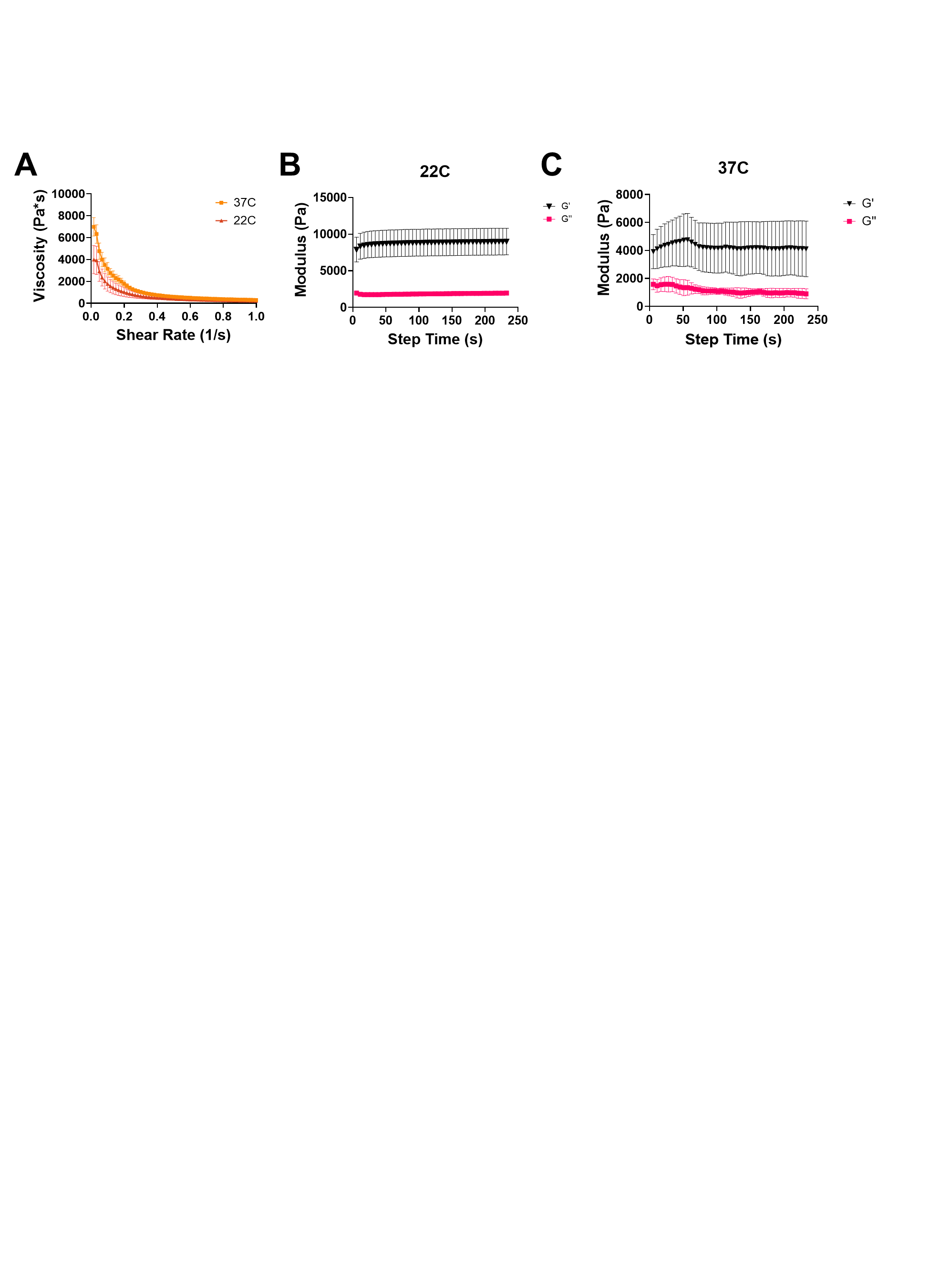
**

*Figure S1. Rheological properties of 20% Pluronic F-127 80% water formulation. Average ± SEM values for viscosity shear sweep (A) and storage and loss modulus data at 22 (B) and 37C (C). N=3.*

*
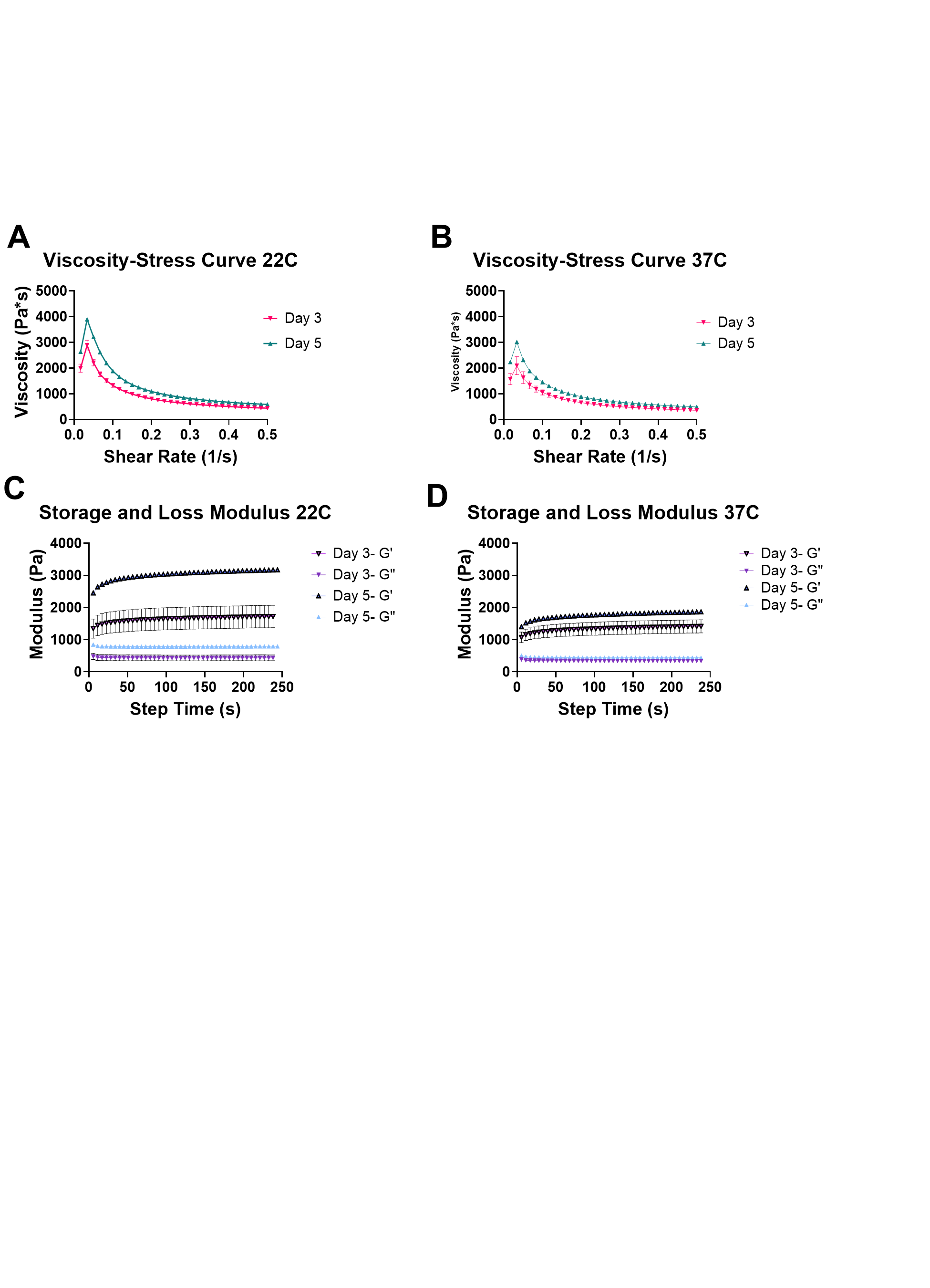
*

*Figure S2. Day 3 and 5 rheology data from one-week stability study. Average ± SEM values viscosity stress curves at 22C (A) and 37C (B) and storage and loss moduli at 22C (C) and 37C (D) for 70% ethanol 10% MC 20% H2O gel stored at room temperature. N=3.*
